# Technical requirements and optimization strategies for home-based teleradiology workstations: a review article

**DOI:** 10.1186/s13244-025-02081-8

**Published:** 2025-09-18

**Authors:** Mustafa S. Alhasan, Ayman S. Alhasan

**Affiliations:** 1 Department of Internal Medicine, College of Medicine, Taibah University, Madinah, Saudi Arabia; 2 Teleradiology Solutions, Ardmore, PA USA; 3 Department of Radiology, King Faisal Specialist Hospital & Research Centre, Madinah, Saudi Arabia

**Keywords:** Teleradiology, Telemedicine, Radiology practice, Computational radiology

## Abstract

**Abstract:**

Teleradiology has advanced from an occasional modality to a cornerstone of modern radiology practice, with the COVID-19 pandemic catalyzing widespread adoption of home-based reading environments. This review synthesizes current literature and expert recommendations on hardware and software optimization for effective home-based teleradiology implementation. Available data indicate 65% of institutions established home workstations during the pandemic, with 74% transitioning routine daytime shifts to internal teleradiology. We reviewed key components of successful remote reading environments, including diagnostic display specifications, environmental controls, ergonomic considerations, computational infrastructure, and network architecture. Evidence suggests that properly configured remote workstations maintain diagnostic performance equivalent to hospital reading rooms while enhancing radiologist satisfaction and productivity. We found that 65% of radiologists reported reduced stress levels when working from home, and 96% observed similar or improved report turnaround times. Essential technical specifications include display luminance standards, ambient lighting levels between 25 and 75 lux, acoustic conditions below 40 decibels, and temperature control within 20–24 °C. Computational requirements include a minimum sustained bandwidth of 50–100 Mbps. We review multi-layered security architectures and workflow integration strategies supporting distributed reading environments. Our review concludes that properly implemented home-based teleradiology is a viable practice model extending specialized expertise across geographic boundaries while promoting radiologist well-being. However, knowledge gaps remain in technical standardization, regulatory harmonization, and long-term clinical outcomes, underscoring the need for further research to support confident, data-driven teleradiology implementation.

**Critical relevance statement:**

This review critically evaluates the technical, ergonomic, and operational requirements for home-based teleradiology, offering evidence-based recommendations that address current practice gaps and support the development of sustainable, high-performance remote reading environments in modern clinical radiology.

**Key Points:**

- Home teleradiology maintains diagnostic quality while improving radiologist well-being; 65% report reduced stress and 96% show similar or improved report turnaround times.
- Optimal implementation requires medical-grade displays, a controlled environment (25–75 lux lighting), 50–100 Mbps bandwidth, and robust security measures.
- Standardization varies across jurisdictions; some countries have protocols, but gaps persist in cross-border teleradiology and long-term outcomes assessment.

**Graphical Abstract:**

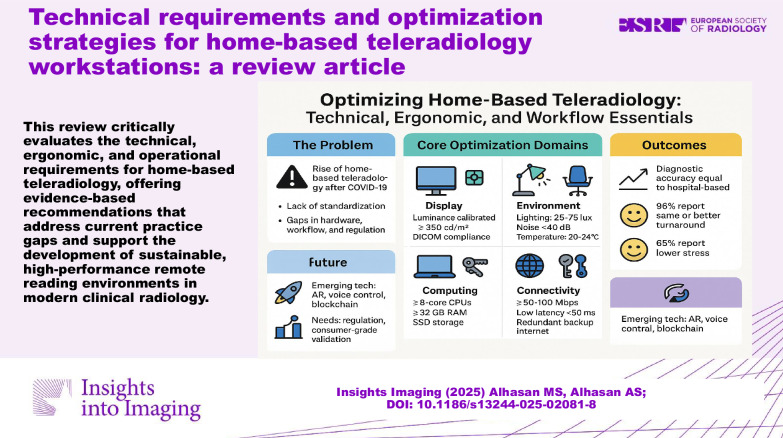

## Introduction

Teleradiology has evolved from addressing after-hours coverage gaps to becoming a cornerstone of modern radiology practice, with 85.6% of U.S. radiologists reporting engagement in remote reading by 2019 [1].

**Fig. 1:**
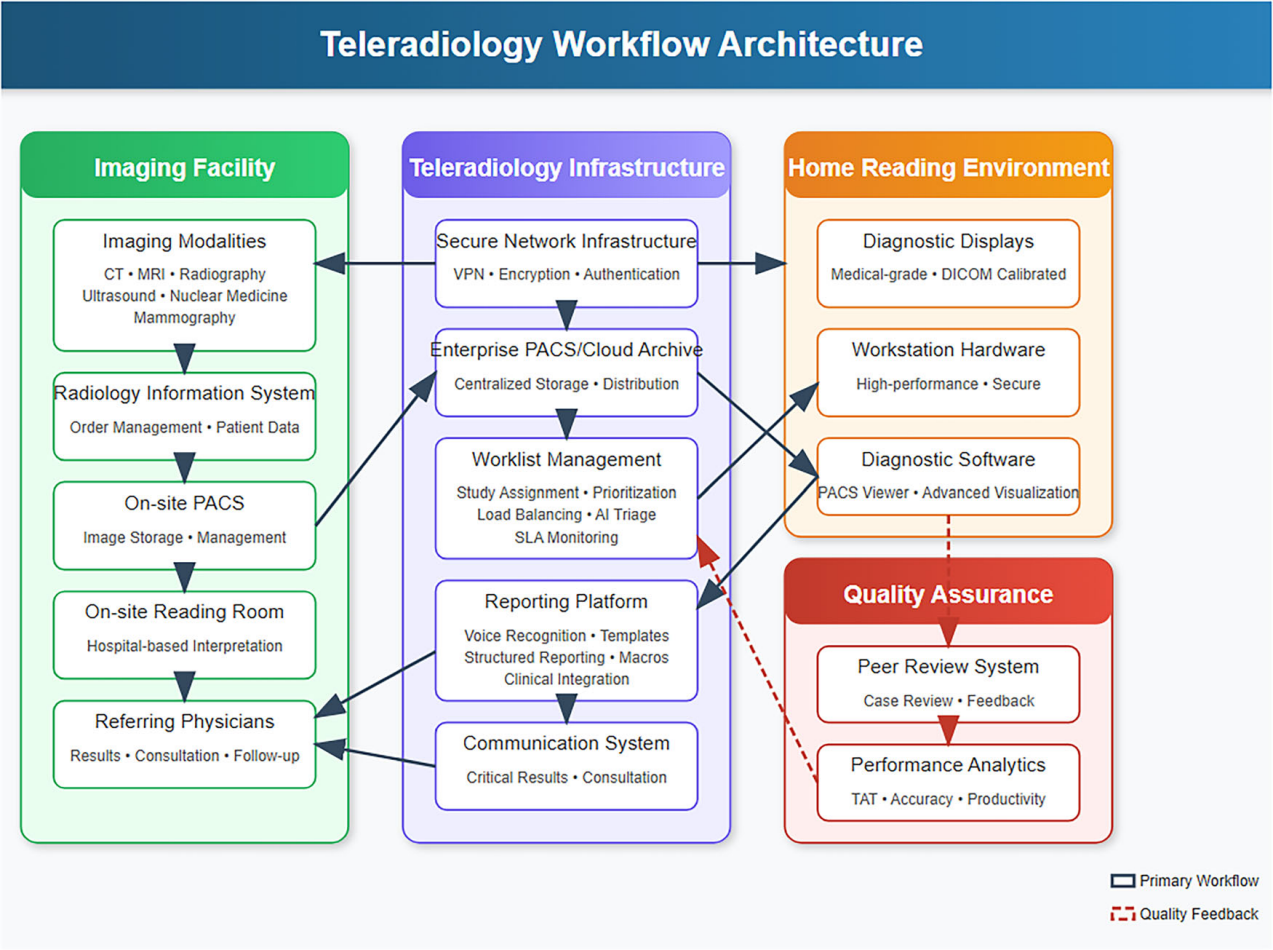
Teleradiology workflow architecture. Complete workflow showing: Imaging Facility (green) with modalities, PACS, and on-site reading; Teleradiology Infrastructure (blue) providing secure connectivity, worklist management, and reporting platforms; Home Reading Environment (orange) with diagnostic displays and workstation hardware; and Quality Assurance framework (red) with peer review and performance monitoring. Arrows indicate workflow direction and feedback loops

The COVID-19 pandemic significantly accelerated the adoption of teleradiology, transforming it from a primarily after-hours or supplementary service into a mainstream radiology practice model (Fig. 1). In response to social distancing measures, healthcare institutions rapidly implemented home workstations to ensure continuity of diagnostic services while safeguarding staff [2]. This abrupt shift exposed both the strengths and limitations of existing teleradiology infrastructure. Academic medical centers and private practices alike faced technical, regulatory, and operational challenges in establishing home-based reading environments capable of maintaining diagnostic quality while promoting radiologist well-being. Survey data reflect this paradigm shift: approximately 65% of institutions deployed home workstations, and 74% transitioned routine daytime shifts to internal teleradiology during the pandemic. Notably, over half of radiologists continued remote work practices even after the easing of acute pandemic restrictions, indicating a sustained change in radiology practice patterns [3].

The sustainability of the shift toward hybrid and home-based practice models hinges on the effective optimization of remote workstations. Unlike the controlled conditions of hospital reading rooms, home-based setups present unique challenges related to hardware specifications, software configurations, network reliability, ergonomic design, and quality assurance protocols. In response, professional organizations have issued guidelines tailored specifically to home-based teleradiology. Notably, the American College of Radiology (ACR), American Association of Physicists in Medicine (AAPM), and Society for Imaging Informatics in Medicine (SIIM) jointly released the *Electronic Practice Standard* in 2022, outlining requirements for display systems, computing hardware, and security infrastructure appropriate for remote diagnostic interpretation [4]. Similarly, the Royal College of Radiologists (RCR) and the Turkish Society of Radiology (TSR) have published technical and operational recommendations to support high-quality home-based radiology reporting [5, 6].

The widespread adoption of remote reading has yielded substantial data on both technical performance and radiologist experience. Empirical evidence indicates that well-configured home workstations can maintain diagnostic accuracy while enhancing radiologist satisfaction and productivity. Approximately 65% of radiologists report reduced stress levels when working from home, and 96% note similar or improved report turnaround times [7]. Nonetheless, notable challenges persist, particularly in the areas of trainee education, team cohesion, and quality assurance in distributed reading environments. The educational impact is especially concerning, with 52% of trainees reporting diminished opportunities for case review and real-time feedback during remote instruction [8]. In this review article, we aim to synthesize current literature and expert perspectives to provide evidence-based guidance for optimizing hardware and software in home-based teleradiology workstations.

## Display technology and image interpretation environment

### Display technology comparison

The diagnostic display is the critical interface between radiological data and the interpreting radiologist, directly influencing detection sensitivity and diagnostic confidence. Medical-grade monitors, specifically engineered for radiologic interpretation, differ markedly from consumer-grade displays in several key dimensions. One of the most important distinctions is compliance with the DICOM Part 14 Grayscale Standard Display Function (GSDF), which ensures accurate grayscale rendering and consistent contrast perception across varying luminance levels [5, 9–12]. Medical-grade displays also maintain strict luminance uniformity, typically with less than 30% variation across the screen, compared to potential variations exceeding 50% in consumer monitors. Additionally, medical displays are designed for long-term calibration stability, with premium models retaining accuracy over thousands of operating hours, whereas consumer displays may exhibit significant drift after just a few hundred hours. These technical differences account for the substantial price gap: medical-grade displays generally range from $5000 to $15,000 per unit, compared to $500–$2000 for high-end consumer monitors. This cost differential underscores why professional guidelines consistently discourage the use of general-purpose displays for primary diagnosis in teleradiology settings [13, 14].

Liquid crystal displays (LCD) with light-emitting diode (LED) backlighting remain the standard for diagnostic imaging, offering proven performance and established quality assurance protocols. [15–17]. While some medical display manufacturers have begun incorporating OLED and mini-LED into diagnostic-grade monitors, professional organizations have adopted a cautious stance, awaiting further validation studies to confirm their equivalence, or superiority, to standard LCD systems for primary diagnostic use (Table 1).

**Table 1:** Essential components of a home-based teleradiology workstation

Component category	General radiography/CT/MRI	Mammography	Nuclear medicine/fusion imaging	Minimum specifications (all modalities)
Display				
Resolution	≥ 3 MP (2048 × 1536)	≥ 5 MP (2560 × 2048)	≥ 3 MP with color calibration	As specified by modality
Luminance	≥ 350 cd/m²	≥ 420 cd/m²	≥ 350 cd/m²	Stable during operational lifetime
Contrast ratio	≥ 350:1	≥ 450:1	≥ 350:1	Measurable using AAPM TG18 test patterns
Pixel pitch	≤ 0.21 mm	≤ 0.17 mm	≤ 0.21 mm	Uniform across display surface
Calibration	DICOM GSDF	DICOM GSDF	DICOM GSDF + color (sRGB)	Initial and quarterly verification
Configuration	**Minimum total display area: 6–10 MP effective viewing** Options: (a) Dual 3–5 MP displays, OR (b) Single large display (≥ 30”, ≥ 6MP) Selection based on workflow, space, and user preference	**Same options, with** ≥ **5 MP primary for mammography**	**Same options + color-capable display for fusion imaging**	**Plus 1 administrative display for all configurations**
Computing hardware				
Processor	Multi-core CPU (≥ 8 cores, ≥ 3.0 GHz)	Same	Same plus specialized processing	Intel i7/i9 or AMD Ryzen 7/9 equivalent
Memory	≥ 32 GB RAM	≥ 32 GB RAM	≥ 64 GB RAM	DDR4-3200 or faster
Storage	Primary: NVMe SSD ≥ 1 TB Secondary: ≥ 2 TB for local caching	Same	Same plus expanded cache ≥ 4 TB for local caching	≥ 3000 MB/s read speed Encrypted at rest
Graphics	Professional GPU, ≥ 8 GB VRAM	Same	Professional GPU, ≥ 16 GB VRAM	NVIDIA RTX/Quadro or AMD Radeon Pro
Network				
Download bandwidth	≥ 100 Mbps sustained	≥ 150 Mbps sustained	≥ 200 Mbps sustained	Business-class service with SLA
Upload bandwidth	≥ 20 Mbps sustained	≥ 30 Mbps sustained	≥ 50 Mbps sustained	Verified through regular testing
Latency	≤ 50 ms to primary systems	Same	Same	≤ 15 ms jitter
Reliability	≥ 99.9% uptime	Same	Same	Redundant connectivity recommended
Security	VPN with AES-256 encryption	Same	Same	Split-tunnel configuration
Environment				
Ambient lighting	25–75 lux at display surface	20–40 lux	25–75 lux with controlled reflections	Indirect, adjustable intensity
Sound level	≤ 40 dB ambient noise	Same	Same	Sound isolation from household
Temperature	20–24 °C (68–75 °F)	Same	Same	Stable during reading sessions
Ergonomics	ANSI/BIFMA compliant chair Monitor height at/below eye level	Same	Same	Height-adjustable desk Adjustable monitor arms
Power protection				
UPS	≥ 15 min runtime at full load	Same	Same	Pure sine wave output
Surge protection	≥ 1500 joule rating	Same	Same	Equipment insurance
Peripherals				
Input devices	Ergonomic keyboard and mouse Optional: programmable keypad	Same	Same plus specialized input	Programmable function keys Customizable macros
Dictation	Noise-canceling microphone Optional: foot pedal	Same	Same	≥ 16-bit/48 kHz audio quality Programmable control

Display configuration should prioritize total effective viewing area and workflow efficiency. Both dual-monitor and single large monitor configurations demonstrate equivalent diagnostic performance when meeting resolution and calibration requirements (ACR Technical Standard). Selection factors include available space, workflow patterns, radiologist preference, and institutional standardization needs

*MP* megapixel, *cd/m²* Candela per square meter, *DICOM GSDF* Digital Imaging and Communications in Medicine Grayscale Standard Display Function, *SLA* service level agreement, *UPS* uninterruptible power supply, *AAPM* American Association of Physicists in Medicine, *NVMe* non-volatile memory express, *SSD* solid state drive, *VRAM* video random access memory, *AES* advanced encryption standard, *ANSI/BIFMA* American National Standards Institute/Business and Institutional Furniture Manufacturer’s Association, *VPN* virtual private network, *GPU* graphics processing unit, *CPU* central processing unit, *RAM* random access memory, *dB* decibel, *TB* terabyte, *GB* gigabyte, *GHz* gigahertz, *Mbps* megabits per second, *ms* milliseconds

### Luminance standards and modality-specific requirements

Luminance specifications for diagnostic displays vary based on imaging modality, reflecting the distinct visual demands of different types of radiologic interpretation. For general radiography, computed tomography (CT), and magnetic resonance imaging (MRI), displays are typically required to maintain a calibrated luminance of at least 350 cd/m², with a minimum black level below 1 cd/m² and a contrast ratio exceeding 350:1. Mammographic interpretation necessitates more stringent performance criteria due to the need for detecting microcalcifications and subtle architectural distortions. Current guidelines recommend a minimum luminance of 420 cd/m² and a display resolution of at least 5 megapixels for mammography [18]. Additionally, nuclear medicine and certain advanced visualization applications benefit from color-capable displays with accurate color rendering, provided they also meet luminance and grayscale standards appropriate for diagnostic interpretation [19, 20].

In home-based teleradiology, modality-specific imaging requirements must be directly reflected in equipment selection and validation protocols. The Royal College of Radiologists (RCR) recommends that remote workstations replicate the display specifications used in on-site environments for equivalent imaging modalities. This guidance emphasizes that home-reporting monitors should not only match hospital-based systems in screen size or appearance, but also in technical parameters such as resolution, luminance, contrast ratio, and calibration status. Similarly, the Turkish Society of Radiology (TSR) advises that home workstations be equipped with displays offering a resolution of at least three megapixels for general radiologic interpretation, and 5 megapixels or higher for mammography, thereby maintaining diagnostic consistency with institutional standards [5, 6].

### Ambient environment control

The interpretation environment surrounding diagnostic displays significantly influences visual perception and diagnostic accuracy. Among environmental variables, ambient lighting is paramount; excessive or improperly directed light can degrade contrast perception through screen glare and pupillary constriction. Professional guidelines recommend maintaining ambient lighting between 25–75 lux at the display surface, substantially lower than typical residential lighting levels, which often exceed 300 lux. This necessitates targeted workspace modifications for home-based teleradiology, such as room-darkening window treatments, indirect lighting fixtures, and matte-finished surfaces to minimize reflective artifacts [21–23].

In addition to lighting, acoustic conditions also affect interpretive performance. Background noise levels below 40 decibels are advised to reduce cognitive distraction and promote sustained focus, particularly during complex case evaluations. Achieving this in a home setting may require sound insulation, dedicated quiet spaces isolated from household activity, or the use of active noise-canceling technologies. Temperature regulation further contributes to optimal cognitive function, with evidence suggesting peak mental performance in environments maintained between 20 and 24 °C (68–75 °F) [24, 25]. Collectively, these environmental elements form an interpretation ecosystem that can either support or undermine diagnostic accuracy, regardless of the quality of the display hardware itself.

## Quality assurance and calibration requirements

Diagnostic displays require quality assurance protocols to maintain consistent performance in distributed teleradiology environments (Table 2). Daily automated calibration using internal photometers ensures DICOM GSDF compliance, while weekly manual verification with external photometers validates luminance stability and contrast ratios. Monthly assessments should evaluate luminance uniformity, with deviations not exceeding 30% across the display surface.

**Table 2:** Teleradiology quality assurance framework

QA component	Requirements	Measurement method	Implementation frequency	Remote-specific considerations
Technical quality assurance				
Display calibration	DICOM GSDF compliance; Luminance 350–450 cd/m²; Contrast ratio ≥ 350:1	AAPM TG18 test patterns; Luminance uniformity measurement	Initial validation plus quarterly recalibration	Remote calibration verification; Home environment lighting assessment; Remote monitoring software
Network performance	Sustained bandwidth meeting modality requirements; Latency < 50 ms; Uptime > 99.9%	Active monitoring of throughput, latency, packet loss; Periodic stress testing	Continuous monitoring with weekly reporting	ISP service level guarantees; Redundant connection availability; Automated alert systems
Workstation validation	Standard enterprise configuration; Performance benchmarking; Security compliance	Benchmark suite execution; Configuration compliance scanning	Initial certification plus monthly validation	Remote management capabilities; Auto-remediation tools; Secure administration channel
Interpretive quality assurance				
Peer review process	Random case selection (5–10% of studies); Blinded review methodology; Standardized scoring system	RADPEER or equivalent methodology; Subspecialty-appropriate case matching	Minimum 5–10 cases per radiologist monthly	Digital peer review platform; Cross-location case distribution; Anonymous reviewer identification
Diagnostic accuracy	Reference standard comparison; Surgical/pathological correlation; Follow-up analysis	Sensitivity/specificity calculation; Positive predictive value assessment	Quarterly analysis of critical findings; Annual comprehensive review	Remote access to clinical outcome data; Integrated feedback mechanisms
Critical result communication	Direct communication protocols; Closed-loop documentation; Escalation pathways	Time to notification measurement; Documentation completeness audit	100% of critical findings tracking; Monthly compliance audit	Remote communication tools; Digital acknowledgment tracking; Multi-channel notification options
Operational quality assurance				
Turnaround time monitoring	Modality-specific targets; Priority-based metrics	Time-stamped workflow tracking; Modality-specific TAT calculation	Continuous monitoring with daily reporting	Remote productivity dashboards; Comparative performance metrics; Environmental factor assessment
Workload distribution	Volume-based assignments; Complexity factor adjustment; Subspecialty alignment	RVU tracking; Complexity-adjusted productivity measurement	Daily workload assessment; Weekly balance review	Remote worklist management tools; AI-assisted complexity scoring; Dynamic assignment algorithms
System availability	99.9% uptime target; Scheduled maintenance windows; Redundancy verification	End-to-end availability measurement; Component-specific uptime calculation	Continuous monitoring with real-time alerting	Remote system monitoring; Distributed availability tracking; Disconnected operation capabilities
Remote-specific quality assurance				
Home environment assessment	Private workspace; Lighting control (25–75 lux); Acoustic management (< 40 dB)	Standardized assessment checklist; Photographic documentation	Initial implementation plus annual recertification	Remote workspace certification; Environmental factor verification; Alternative location requirements
Remote radiologist well-being	Virtual engagement strategies; Burnout prevention protocols	Standardized well-being surveys; Productivity pattern analysis	Quarterly well-being assessment	Virtual community development; Isolation mitigation protocols; Structured connection opportunities
Communication effectiveness	Virtual communication platforms; Structured interaction protocols	Communication frequency measurement; Satisfaction survey data	Monthly assessment	Multi-channel communication tools; Virtual presence technologies; Backup communication pathways

*AAPM* American Association of Physicists in Medicine, *AI* artificial intelligence, *DICOM* Digital Imaging and Communications in Medicine, *GSDF* grayscale standard display function, *ISP* internet service provider, *RADPEER* radiology peer review system, *RVU* relative value unit, *TAT* turnaround time, *TG* task group

Acceptance testing must establish baseline performance characteristics, including maximum luminance output (≥ 350 cd/m² for general radiology, ≥ 420 cd/m² for mammography), contrast ratios, and spatial resolution compliance with manufacturer specifications. Previous evidence has demonstrated significant performance variations between monitor technologies, focusing on the importance of standardized QA protocols tailored to specific display characteristics [26]. Environmental monitoring should document ambient lighting levels (25–75 lux) and viewing conditions to ensure the best diagnostic performance.

Regulatory compliance requires extensive and detailed documentation of calibration activities, performance measurements, and corrective actions. For teleradiology applications, remote QA execution may utilize video documentation and electronic monitoring systems to satisfy accreditation requirements while maintaining the flexibility of distributed reading environments.

## Ergonomics and human factors engineering

### Musculoskeletal disorder prevention

Radiologists are at considerable risk for developing work-related musculoskeletal disorders (MSDs) due to prolonged static postures, repetitive motions, and suboptimal workstation configurations. Studies report that up to 60% of radiologists experience symptoms of musculoskeletal discomfort, most commonly affecting the neck, lower back, and upper extremities [27]. Effective prevention begins with ergonomically designed seating [28–30].

**Fig. 2:**
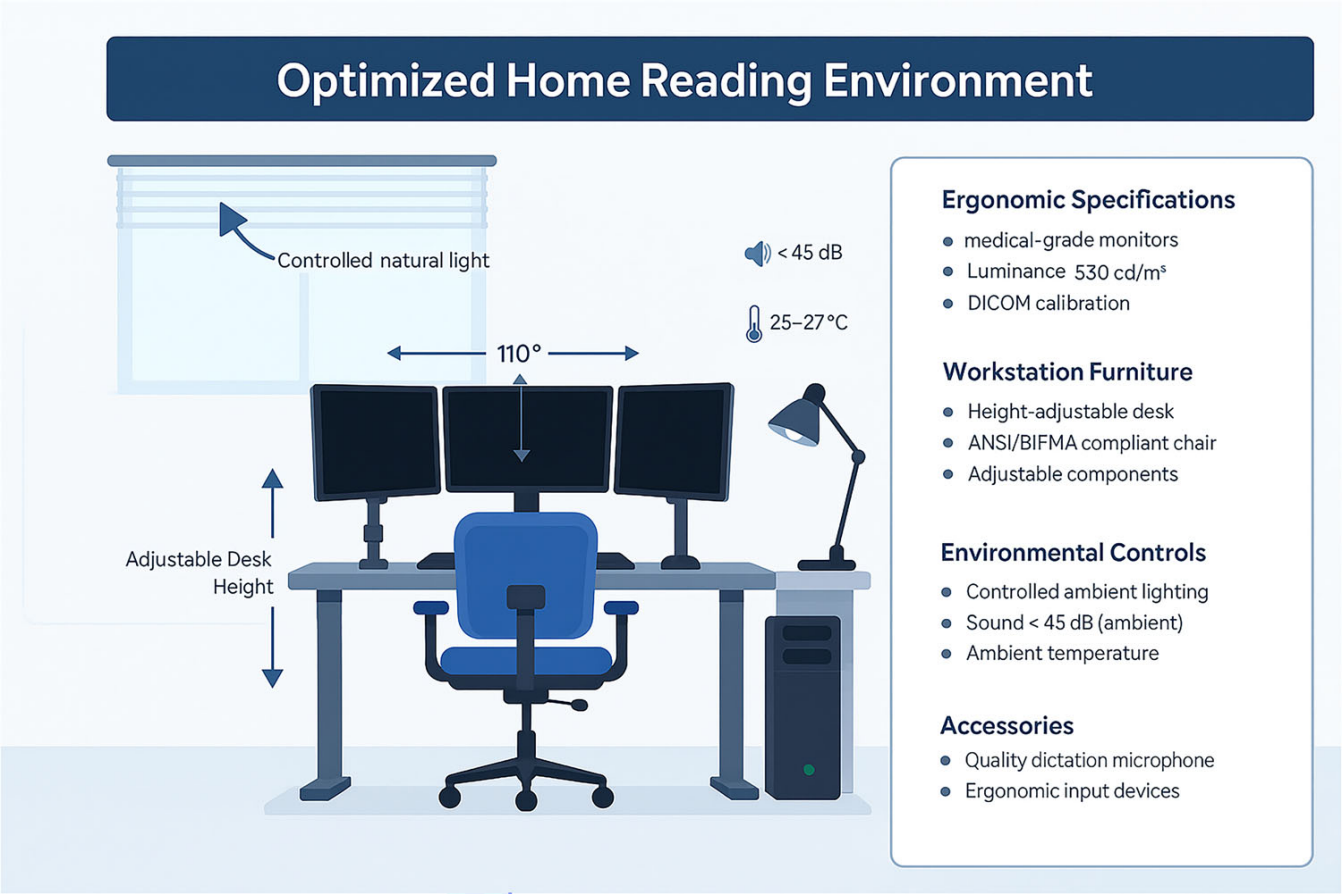
Optimized home reading environment illustration

Workstation layout is another critical factor. Height-adjustable desks that accommodate both sitting and standing postures have been shown to reduce static spinal loading. Evidence supports alternating between sitting and standing every 30–45 min to maximize musculoskeletal benefit without impairing workflow efficiency or diagnostic accuracy [31, 32]. Monitor positioning has a direct impact on cervical strain. Optimal setup involves placing the top edge of the primary display at or just below eye level and maintaining a viewing distance of approximately 20–40 inches from the user [33]. When using multiple displays, monitors should be arranged to minimize head rotation, typically through curved or angled configurations that preserve consistent viewing distances across all screens (Fig. 2).

### Visual fatigue mitigation strategies

One widely supported intervention is the 20-20-20 rule: looking at an object 20 feet away for 20 s every 20 min. This technique has been shown to alleviate accommodation fatigue and can be effectively integrated into the workflow through scheduled break reminders [34, 35].

## High-performance computing infrastructure

### Computational requirements analysis

The computing hardware supporting teleradiology workstations must provide consistent, high-performance capabilities across a broad range of imaging modalities and advanced visualization tasks. Hardware requirements vary by modality, with basic radiography and limited CT interpretation demanding relatively modest resources, whereas applications involving multiplanar reconstruction, volumetric rendering, and fusion imaging require substantially greater processing power [36].

### Graphical processing units acceleration benefits

Graphics processing units (GPUs) play an increasingly critical role in radiologic interpretation, particularly in the context of advanced visualization and artificial intelligence (AI) applications. Many modern PACS viewers leverage GPU acceleration to enhance performance for resource-intensive tasks such as three-dimensional rendering, maximum intensity projections, and real-time volumetric manipulation. Performance benchmarking has shown that GPU-accelerated rendering can improve frame rates by 30–600% compared to CPU-only processing, with the greatest benefits observed in complex operations like cinematic rendering and volume visualization [37].

### System redundancy engineering

Redundancy engineering is essential to mitigate potential points of failure, starting with power supply reliability. Uninterruptible power supply (UPS) systems capable of supporting the entire workstation for at least 15–30 min are considered a minimum standard for teleradiology, enabling graceful shutdown during outages and protecting against power fluctuations commonly encountered in residential settings [38].

## Connectivity and network architecture

### Bandwidth requirements

Network connectivity is a critical component of teleradiology infrastructure, serving as the primary link between remote radiologists and institutional imaging systems. Bandwidth requirements are influenced by study complexity, workflow intensity, and daily interpretative volume. Download bandwidth directly affects image retrieval speed, with current guidelines recommending a minimum sustained rate of 50–100 Mbps for general teleradiology applications, and over 200 Mbps for high-volume or subspecialty workflows involving large datasets, such as multiphase CT, breast tomosynthesis, or dynamic cardiac MRI [39].

Upload bandwidth demands have also increased, driven by the need for robust bidirectional communication. Sustained upload speeds of 20–50 Mbps are recommended to support smooth video conferencing, screen sharing during interdisciplinary consultations, and timely transmission of finalized reports. Importantly, these figures represent minimum operational thresholds rather than ideal targets. High-performing teleradiology environments often exceed these benchmarks to ensure consistent performance during peak usage periods and to accommodate the growing size and complexity of imaging datasets [39–41].

### Latency management strategies

While bandwidth metrics quantify data transfer capacity, latency measurements are equally critical for teleradiology performance, particularly in tasks requiring real-time interactivity. Latency, commonly measured as round-trip time between the radiologist’s workstation and institutional systems, directly affects responsiveness during operations such as window/level adjustments, image scrolling, and region-of-interest manipulation. For optimal performance, round-trip latency should ideally remain below 50 milliseconds to ensure fluid and responsive image interaction [42].

Latency management often involves network route optimization, which aims to minimize physical distance and the number of network hops between endpoints. Techniques such as Border Gateway Protocol (BGP) routing controls, traffic engineering, and strategic peering arrangements can reduce latency by 20–40% compared to default internet paths. However, implementing such optimizations typically requires enterprise-level network management capabilities, which may not be feasible for individual practitioners operating from home environments [43].

### Network security architectures

Security requirements for teleradiology connections must strike a careful balance between robust protection of patient data and operational efficiency to support timely clinical access. Modern security architectures employ a layered, defense-in-depth approach, leveraging multiple technologies rather than relying on a single point of protection. Virtual Private Network (VPN) solutions, including internet protocol security (IPsec), secure sockets layer/transport layer security, and datagram transport layer security (DTLS) protocols, create encrypted tunnels between teleradiology workstations and institutional systems, safeguarding data in transit across public or unsecured networks [44–46].

Authentication frameworks have evolved significantly, moving beyond basic password-based systems to more advanced multi-factor authentication (MFA) models. These frameworks incorporate combinations of knowledge factors (e.g., passwords, PINs), possession factors (e.g., smart cards, hardware tokens, registered mobile devices), and biometric factors (e.g., fingerprint scans, facial recognition, behavioral biometrics). The goal is to enhance security without impairing usability, acknowledging that overly burdensome authentication processes may encourage workarounds that ultimately compromise system integrity [47–51].

## Software optimization

### Distributed architecture models

The technological architecture underlying teleradiology has evolved significantly, from basic remote viewing of centralized image repositories to advanced distributed systems engineered for enhanced performance, scalability, and reliability across diverse environments. Traditional thick-client architectures, in which teleradiologists operate full-featured workstation software connected to centralized PACS databases, continue to provide strong rendering performance and support for advanced visualization. However, these benefits come with increased complexity in terms of deployment, configuration, and ongoing maintenance.

More recently, cloud-native PACS architectures have emerged as a transformative development in distributed imaging platforms. These systems leverage global cloud infrastructure to offer improved accessibility, scalability, and resilience, often outperforming traditional on-premises solutions. Built on microservices architectures, they break down monolithic PACS functionality into modular components that can be independently deployed and dynamically scaled based on workload demands. For teleradiology, this architecture presents distinct advantages, including geographically distributed processing to reduce latency, elastic scaling during peak workloads, and built-in redundancy via multi-region deployments [52, 53].

### Prefetching algorithms

Intelligent prefetching mechanisms play a crucial role in enhancing teleradiology performance by proactively retrieving relevant prior studies and related examinations before radiologists explicitly request them. This approach effectively masks network latency by aligning data retrieval with workflow timing. Rule-based prefetching systems apply static criteria, such as anatomical region, imaging modality, and temporal proximity, to identify pertinent comparison studies. These are typically fetched automatically when a new case is added to the radiologist’s worklist, provided they meet predefined relevance thresholds [54].

In teleradiology implementations, the prefetching strategy significantly influences both interpretation efficiency and overall network usage. Aggressive prefetching maximizes study availability and minimizes delays during image interpretation but may result in the retrieval of many studies that are ultimately unused, leading to unnecessary bandwidth consumption. Conversely, conservative prefetching reduces nonessential data transfers but may introduce workflow delays when radiologists request prior studies not previously retrieved. Optimal prefetching strategies must therefore balance performance gains with bandwidth efficiency, particularly in distributed or bandwidth-limited environments [54, 55].

Modern PACS architectures increasingly leverage real-time streaming technologies that have largely superseded traditional prefetching approaches. Progressive JPEG 2000 and adaptive streaming protocols enable dynamic image delivery based on bandwidth availability and user interaction patterns. These streaming implementations provide immediate image access while minimizing bandwidth waste, particularly beneficial for teleradiology, where network resources may be limited. However, hybrid approaches combining intelligent prefetching of high-priority studies with streaming capabilities for on-demand access represent the current state-of-the-art for optimized teleradiology performance [56–59].

### Voice recognition technology comparison

Modern speech recognition systems achieve over 95% accuracy for radiological dictation [60–62]. Cloud-based solutions offer superior accuracy but introduce 200 ms to 500 ms latency compared to local processing [63–68].

### Structured reporting implementation strategies

In teleradiology applications, structured reporting offers distinct advantages, including improved consistency across distributed interpreters, streamlined quality monitoring, and reduced dictation burden, which may help mitigate speech recognition challenges in variable acoustic environments [69]. Effective implementations prioritize template harmonization across all reading locations to ensure uniform report structure and terminology, regardless of where the interpretation is performed. At the same time, systems must allow for location-specific modifications to accommodate the unique preferences or clinical requirements of particular facilities or service lines [70].

## Security and compliance framework

### Multi-layered security architecture

Effective teleradiology security necessitates coordinated protection across multiple technical layers, system components, and potential attack vectors, rather than reliance on isolated security measures. Modern security architectures adopt a defense-in-depth strategy, emphasizing redundant safeguards to ensure that the compromise of any single control does not lead to total system exposure. At the foundation of this architecture is physical security, which includes controls that restrict unauthorized access to teleradiology workstations and supporting infrastructure, forming the baseline upon which higher-level security measures are built [71, 72].

System security combines workstation hardening (restricted privileges, endpoint protection) with application-level controls including secure authentication and comprehensive threat protection (Table 3).

**Table 3:** Overview of teleradiology security framework

Security domain	Implementation requirements	Regulatory requirements	Risk mitigation strategies	Validation methods	Implementation priority	Implementation timeline	Responsible parties	Monitoring frequency	Cost impact	Breach impact
Physical security: workstation environment	Dedicated lockable space; Access restrictions; Privacy screens (≥ 60° angle); Environmental monitoring	HIPAA §164.310(a)(1); GDPR Art. 32; ISO 27001 A.11	Room access logs; Clear screen policy; Authorized personnel documentation; Clean desk protocols	Physical security audit (quarterly); Documented ownership; Photographic setup verification	Required	Implementation Phase 1 (0–30 days)	Radiologist; IT Security	Quarterly validation	Low	High
Physical security: media handling	Encrypted media (AES-256); Secure disposal (DoD 5220.22-M); Asset tracking; No unencrypted PHI	HIPAA §164.310(d)(1); HITECH §13402; ISO 27001 A.8.3	Media inventory management; Destruction certification; DLP scanning; Media handling policy	Destruction verification; Quarterly compliance check; DLP reports	Required	Implementation Phase 1 (0–30 days)	IT Operations; Security Team; Radiologist	Monthly scanning; Quarterly audit	Low-Medium	High
Network security: perimeter protection	Enterprise-grade NGFW; IDS/IPS with medical protocols; Network segmentation; Dedicated teleradiology VLAN	HIPAA §164.312(e)(1); NIST SP 800-41; ISO 27001 A.13.1	Boundary protection devices; Default-deny rules; Regular rule review; Protocol filtering	Vulnerability scanning (monthly); Penetration testing (annual); Configuration review	Critical	Implementation Phase 1 (0–30 days)	Network Security Team; IT Operations	Continuous monitoring; Monthly reporting	Medium-High	Severe
Network security: data transmission	TLS 1.3 or IPsec with PFS; AES-256 minimum; Certificate authentication; Automated certificate management	HIPAA §164.312(e)(1); FIPS 140-2/3; NIST SP 800-52r2	Protocol downgrade prevention; Certificate monitoring; Cryptographic assessment; Key management	Encryption validation; TLS configuration audit; Packet capture analysis	Critical	Implementation Phase 1 (0–30 days)	Network Security Team; Security Architecture	Weekly certificate monitoring; Quarterly configuration review	Medium	Severe
Network security: remote access	Split-tunnel VPN (clinical traffic); MFA (NIST AAL2+); Context-aware policies; Detailed connection logging	HIPAA §164.312(a)(2)(iv); NIST SP 800-63B; ISO 27001 A.6.2	Connection time limits; Geo-restrictions; Device posture checking; Access alerting	VPN configuration audit; Auth log review; Connection pattern analysis	Critical	Implementation Phase 1 (0–30 days)	Identity Team; Network Security	Daily log review; Monthly VPN audit	Medium-High	Severe
System security: endpoint protection	EDR/XDR with behavioral analysis; Application allowlisting; Vulnerability management; Privileged access controls	HIPAA §164.308(a)(5)(ii)(B); NIST SP 800-53 SI-7; ISO 27001 A.12.2	BYOD prohibition; Centralized management; Admin restriction; Security baselines	Bi-weekly vulnerability scanning; Configuration audits; Privilege testing	Required	Implementation Phase 2 (31–60 days)	Endpoint Security Team; IT Operations	Daily EDR monitoring; Bi-weekly scans	High	High
System security: patch management	Critical patches < 14 days; Monthly maintenance; Pre-deployment testing; Automated compliance	HIPAA §164.308(a)(5)(ii)(B); NIST SP 800-40r4; ISO 27001 A.12.6	Vulnerability prioritization; Exception process; Legacy isolation; Compensating controls	Patch compliance reports; Remediation tracking; Exception documentation	Required	Implementation Phase 2 (31–60 days)	IT Operations; Security Team	Weekly patch status; Monthly compliance review	Medium	High
System security: device hardening	DISA STIG/CIS benchmarks; Disabled services; Disk encryption; Local firewall	HIPAA §164.308(a)(5)(ii)(D); NIST SP 800-70; ISO 27001 A.14.1.3	Custom security baselines; System snapshots; Boot protection; FIPS 140-2 encryption	Configuration scans; Baseline audits; Benchmark scoring	Required	Implementation Phase 2 (31–60 days)	System Administrators; Security Engineers	Quarterly baseline verification	Medium	High
Application security: authentication	Federated identity (SAML/OIDC); Role-based access; 15–30 min timeouts; Unique identification	HIPAA §164.312(a)(1); NIST SP 800-63 C; ISO 27001 A.9.2	Password complexity; Login monitoring; Account lockout; Privileged monitoring	Auth log analysis; Credential storage tests; Session review	Critical	Implementation Phase 1 (0–30 days)	Identity Team; Application Security	Daily monitoring; Monthly audit	Medium-High	Severe
Application security: PACS/RIS	Current application versions; Vendor security certification; API validation; Third-party assessment	HIPAA §164.308(b)(1); FDA Cybersecurity Guidance; ISO 27001 A.14.2	Vendor security questionnaire; Pen-testing approval; Compensating controls	Vendor assessment; Vulnerability scanning; Configuration review	Critical	Implementation Phase 2 (31–60 days)	Radiology IT; Vendor Management	Quarterly security review	High	Severe
Application security: mobile devices	Container technology; Remote wipe; Camera/microphone controls; Restricted storage	HIPAA §164.310(b); NIST SP 800-124; ISO 27001 A.6.2.1	App whitelisting; Device encryption; Mobile DLP; Screen capture prevention	Mobile security audit; Container isolation tests; Data leakage testing	Recommended	Implementation Phase 3 (61–90 days)	Mobile Security Team; End User Computing	Monthly compliance check	Medium	Medium-High
Data security: classification	PHI identification/tagging; Automated discovery; Minimal necessary principle; Data flow mapping	HIPAA §164.502(b); GDPR Art. 5(1)(c); ISO 27001 A.8.2	DLP rules; Data minimization; Privacy by design; De-identification when possible	Classification audit; Flow mapping; DLP testing; Accuracy validation	Required	Implementation Phase 2 (31–60 days)	Data Security Team; Privacy Officer	Quarterly assessment	Medium	High
Data security: protection	Database encryption (TDE/field); Key management; Data masking; DLP for all states	HIPAA §164.312(a)(2)(iv); GDPR Art. 32(1)(a); ISO 27001 A.10.1	Key rotation; Secure escrow; Hardware security modules; Duty separation	Encryption audit; Key management review; Protection testing	Critical	Implementation Phase 2 (31–60 days)	Data Security; Encryption Team	Monthly key validation; Quarterly audit	High	Severe
Data security: retention	Automated post-report purging; Study-specific policies; Deletion verification; Exception workflow	HIPAA §164.310(d)(2)(i); GDPR Art. 5(1)(e); ISO 27001 A.18.1.3	Schedule enforcement; Cryptographic erasure; Media sanitization; Storage monitoring	Deletion verification; Compliance review; Storage scanning	Required	Implementation Phase 3 (61–90 days)	Records Management; Legal; IT Storage	Weekly purge validation; Monthly compliance	Medium	High
Operational security: audit logging	Centralized SIEM; Tamper-evident logs; ≥ 1 year retention; Access/activity/system logs	HIPAA §164.312(b); NIST SP 800-92; ISO 27001 A.12.4	Correlation analysis; Automated alerting; Integrity validation; Access monitoring	Coverage verification; Integrity testing; Alert effectiveness	Required	Implementation Phase 2 (31–60 days)	Security Operations; Compliance	Daily log review; Monthly integrity check	High	High
Operational security: monitoring	24/7 security monitoring; Behavioral anomaly detection; IOC monitoring; Activity tracking	HIPAA §164.308(a)(1)(ii)(D); NIST SP 800-137; ISO 27001 A.12.7	Behavior profiling; Threshold alerts; Anomaly detection; Real-time monitoring	Detection capability tests; False positive analysis; Response timing	Recommended	Implementation Phase 3 (61–90 days)	Security Operations Center; CIRT	Continuous monitoring; Weekly review	High	Medium-High
Operational security: incident response	IR playbooks; Escalation procedures; Breach notification process; Annual exercises	HIPAA §164.308(a)(6); HITECH §13402; ISO 27001 A.16.1	Incident classification; Evidence preservation; Forensic capability; Communication	Quarterly tabletop exercises; Annual simulation; Post-incident reviews	Required	Implementation Phase 2 (31–60 days)	Incident Response Team; Legal	Quarterly validation exercises	Medium-High	High
User security: awareness	Initial/annual training; Phishing simulations; Role-specific training; Policy acknowledgment	HIPAA §164.308(a)(5); GDPR Art. 32(4); ISO 27001 A.7.2.2	Targeted role training; Microlearning; Security champions; Knowledge assessment	Completion audits; Assessment scores; Simulation metrics	Required	Implementation Phase 3 (61–90 days)	Security Awareness; HR	Monthly phishing tests; Quarterly training	Low-Medium	Medium-High
User security: remote work policies	Acceptable use policy; Home network requirements; Work/personal separation; Environmental standards	HIPAA §164.310(b); NIST SP 800-46; ISO 27001 A.6.2.2	Policy acknowledgment; Remote certification; Environment verification; Agreements	Policy compliance audit; Environment checks; Usage monitoring	Required	Implementation Phase 3 (61–90 days)	HR; Security Policy Team; Teleradiology Director	Annual policy refresh; Quarterly checks	Low	Medium-High
Vendor security: assessment	Vendor security questionnaires; Contract security requirements; Risk rating methodology; Right-to-audit clauses	HIPAA §164.308(b); HITECH §13401; ISO 27001 A.15.1	Security SLAs; Risk-based assessment; Security incident reporting; Key personnel vetting	Initial assessment; Annual review; Security incident analysis	Required	Implementation Phase 2 (31–60 days)	Vendor Management; Procurement; Security	Initial then annual reassessment	Medium	High
Compliance management: documentation	Security policies; Technical standards; Procedures; Guidelines tailored for teleradiology	HIPAA §164.316; GDPR Art. 24; ISO 27001 A.5.1	Document management system; Version control; Approval workflows; Accessibility	Document currency audit; Implementation verification; Effectiveness review	Required	Implementation Phase 1 (0–30 days)	Compliance Team; Security Policy	Annual review; Quarterly spot checks	Low-Medium	Medium-High
Risk management: assessment	Asset inventory; Risk analysis methodology; Threat modeling; Vulnerability management program	HIPAA §164.308(a)(1)(ii)(A); NIST SP 800-30; ISO 27001 A.8.1	Risk register; Mitigation planning; Residual risk acceptance; Executive reporting	Quarterly risk review; Annual comprehensive assessment; Gap analysis	Required	Implementation Phase 1 (0–30 days)	Risk Management; Security Leadership	Quarterly risk review; Daily vulnerability tracking	Medium	High
Business continuity: teleradiology	DR planning; Backup interpretation capabilities; Alternative workstation access; Communication planning	HIPAA §164.308(a)(7); NIST SP 800-34; ISO 27001 A.17	Redundant connectivity; Alternative reading locations; Cloud-based backup PACS; Prioritization	Annual DR testing; Backup capability verification; RTO/RPO validation	Required	Implementation Phase 3 (61–90 days)	Business Continuity; Radiology Operations	Annual full test; Quarterly component tests	High	Severe

*AAL2+* Authentication Assurance Level 2 or higher, *AES* advanced encryption standard, *API* application programming interface, *BYOD* bring your own device, *CIS* Center for Internet Security, *CIRT* Computer Incident Response Team, *DISA STIG* Defense Information Systems Agency Security Technical Implementation Guide, *DLP* data loss prevention, *DR* disaster recovery, *EDR/XDR* endpoint/extended detection and response, *FIPS* Federal Information Processing Standards, *GDPR* General Data Protection Regulation, *HIPAA* Health Insurance Portability and Accountability Act, *HITECH* Health Information Technology for Economic and Clinical Health Act, *IDS/IPS* intrusion detection/prevention system, *IOC* indicators of compromise, *IPsec* Internet Protocol Security, *IR* incident response, *ISO* International Organization for Standardization, *MFA* multi-factor authentication, *NGFW* next-generation firewall, *NIST* National Institute of Standards and Technology, *OIDC* OpenID Connect, *PFS* perfect forward secrecy, *PHI* protected health information, *RIS* Radiology Information System, *RPO/RTO* recovery point objective/recovery time objective, *SAML* security assertion markup language, *SIEM* Security information and event management, *SLA* service level agreement, *TDE* transparent data encryption, *TLS* transport layer security, *VLAN* virtual local area network

### Patient data protection strategies

Protecting patient information in teleradiology workflows requires robust, structured, and context-specific strategies that extend beyond generic security controls to address the unique properties of medical imaging data [73]. One foundational principle is data minimization, which restricts the transmission of personal health information to only those elements essential for diagnostic interpretation. Advanced implementations often employ partial anonymization, preserving clinically relevant identifiers (e.g., age, gender, scan date) while stripping extraneous personal details, thereby reducing the privacy impact in the event of a security breach [74].

## Practical implementation guide

Successful teleradiology implementation requires structured decision-making regarding scope, ranging from limited emergency coverage to comprehensive enterprise deployment, with phased approaches recommended (Table 4).

**Table 4:** Teleradiology implementation models: strategic considerations and relative cost framework

Implementation component	Academic medical center	Community hospital network	Subspecialty practice	Private teleradiology service	Key considerations	Risk factors
Technical infrastructure						
Display systems	High investment priority Medical-grade displays essential Multiple modality support	Medium-High investment Standard diagnostic quality Dual-modality optimization	Highest investment priority Subspecialty-specific requirements Premium calibration needs	Medium investment Standardized enterprise solution Volume-based procurement	Cannot compromise on diagnostic quality. Medical-grade displays required for primary interpretation. Modality-specific requirements must be met.	Technology obsolescence without proper planning. Inadequate specifications reducing diagnostic confidence.
Computing hardware	High performance requirements Multi-tasking capabilities Advanced visualization support	Medium performance requirements Standard clinical workflows Reliable operation priority	High performance requirements Specialized processing needs Advanced analytics support	Medium performance requirements Standardized configurations Proven reliability focus	Hardware must meet minimum performance specifications for expected study volumes. Future-proofing considerations important.	Hardware failure without redundancy. Insufficient performance affecting workflow efficiency.
Network infrastructure	Enterprise-grade connectivity Redundancy essential Institutional integration	Business-class service Multiple site coordination Reliability priority	Specialized optimization High-bandwidth requirements Low-latency critical	Standardized deployment Proven connectivity models Service level agreements	Bandwidth and latency directly impact user experience. Redundancy critical for clinical operations.	Connectivity failures disrupting patient care. Inadequate bandwidth causing workflow bottlenecks.
Security framework	Comprehensive enterprise security Multi-layer protection Institutional compliance	Standard healthcare security Proven security models Regulatory compliance	Specialized security needs Data protection priority Audit trail requirements	Enterprise security framework Standardized implementation Comprehensive monitoring	Must meet all applicable regulatory requirements. Security cannot be compromised.	Regulatory penalties from inadequate security. Data breaches affecting institutional reputation.
Operational considerations						
Quality assurance	Comprehensive academic protocols Peer review integration Teaching requirements	Standard clinical protocols Quality monitoring systems Performance tracking	Subspecialty-focused protocols Specialized metrics Expert review processes	Standardized quality framework Automated monitoring systems Performance benchmarking	QA standards must match on-site interpretation quality. Continuous monitoring essential.	Quality compromise affecting patient outcomes. Inconsistent performance across locations.
Training and support	Advanced continuing education Research integration Trainee supervision	Standard continuing education Clinical workflow focus User support priority	Subspecialty development Advanced techniques Expert consultation	Standardized training programs Efficient support models User competency focus	Training must address both technical and clinical components. Ongoing support critical.	Knowledge gaps causing operational inefficiency. Inadequate support affecting user adoption.
Workflow integration	Complex academic workflows Research coordination Teaching integration	Standard clinical workflows Multi-site coordination Efficiency optimization	Specialized workflows Expert consultation Integration complexity	Optimized production workflows Standardized processes Efficiency maximization	Workflow integration affects user acceptance and efficiency. Change management critical.	Workflow disruption reducing productivity. Poor integration affecting user satisfaction.
Implementation models						
Scope definition	Hybrid academic/clinical model Research support requirements Educational integration	Clinical service focus Multi-site coverage Emergency support	Subspecialty expertise distribution Expert consultation model Geographic expansion	Production optimization Volume-based efficiency Service scalability	Scope must align with institutional goals and capabilities. Phased approach often optimal.	Scope creep affecting project success. Unrealistic expectations causing implementation failure.
Timeline expectations	24–36 months comprehensive implementation Complex requirements Extended validation	18–24 months standard implementation Proven approaches Focused deployment	24–30 months specialized implementation Custom requirements Expert validation	12–18 months standardized implementation Proven methodologies Efficient deployment	Realistic timelines critical for success. Adequate testing and validation time required.	Rushed implementation compromising quality. Extended timelines affecting institutional support.
Success metrics	Academic productivity measures Teaching effectiveness Research integration Quality improvements	Clinical efficiency measures Service quality metrics User satisfaction Cost effectiveness	Subspecialty expertise metrics Expert consultation quality Geographic reach Clinical outcomes	Production volume metrics Service efficiency Quality consistency Cost optimization	Metrics must align with implementation goals. Regular monitoring and adjustment needed.	Inappropriate metrics misguiding decisions. Lack of measurement preventing optimization.
Strategic benefits						
Operational flexibility	Enhanced scheduling flexibility Geographic coverage expansion Improved work-life balance Staff retention benefits	Multi-site service optimization After-hours coverage Emergency response Geographic expansion	Subspecialty expertise distribution Expert consultation availability Improved service reach Enhanced recruitment	Service scalability Production optimization Geographic flexibility Cost efficiency	Benefits realization requires proper implementation and management. Quantification varies by system.	Benefit overestimation affecting ROI. Implementation quality determining actual benefits.
Competitive advantage	Academic excellence maintenance Recruitment enhancement Research capabilities Service differentiation	Service territory expansion Competitive positioning Market leadership Improved efficiency	Subspecialty expertise positioning Market differentiation Expert service delivery Competitive recruitment	Service efficiency optimization Market expansion Cost competitiveness Production scalability	Competitive advantages require sustained execution and quality maintenance.	Competitive threats from poor execution. Market changes affecting advantage sustainability.
Implementation scale						
Small scale (1–5 users)	Limited economies of scope Pilot program potential Research focus	Limited optimization potential Emergency coverage focus	Specialized focus advantage Niche service development	Generally not viable Fixed cost challenges	Requires careful scope management to achieve positive outcomes. Often serves as proof of concept.	Insufficient scale for full benefit realization. High per-user costs.
Medium scale (6–15 users)	Balanced approach Operational efficiency Educational integration	Optimized clinical model Multi-site integration	Specialty service expansion Expert network development	Viable service model Standard deployment	Ideal scale for many practice environments. Balanced approach with significant benefit potential.	Change management challenges without proper planning. Complexity requiring structured approach.
Large scale (16+ users)	Enterprise optimization Multi-specialty coordination Transformative potential	Multi-site integration comprehensive deployment	Multi-specialty coordination Complex service networks	Scalable enterprise model Maximum efficiency	Maximum benefit realization through comprehensive deployment. Requires enterprise project management.	Implementation complexity requiring phased approach. Coordination challenges across multiple sites.

Regional considerations: Cost relationships and implementation considerations based on developed healthcare markets. Significant regional variations expected in absolute costs, regulatory requirements, reimbursement models, and productivity benefits. All operational considerations listed per workstation unless otherwise specified. Institutional costs vary significantly based on scale and integration requirements

*QA* quality assurance, *ROI* return on investment, *SLA* service level agreement

Successful teleradiology deployment requires comprehensive checklists covering technical infrastructure (workstation specifications, connectivity validation, security implementation, environmental assessment) and administrative readiness (credential verification, billing integration, scheduling planning, support infrastructure) to ensure operational readiness.

## Conclusions

Literature review and real-world implementation experience demonstrate that properly implemented home-based teleradiology achieves diagnostic performance equivalent to on-site interpretation while enhancing radiologist satisfaction and extending subspecialty expertise across geographic boundaries. Technical evidence supports high-quality remote interpretation when display technologies meet ACR/AAPM guidelines, despite that mammography and subtle abnormality detection may benefit from controlled reading room conditions.

Significant knowledge gaps persist, including a lack of consensus on network performance specifications, incomplete validation protocols for consumer-grade hardware, and insufficient calibration standards for specialized applications. Regulatory gaps include limited standardization in multi-jurisdictional licensure models and underdeveloped reimbursement frameworks for distributed care. Future studies require detailed, structured methodologies addressing technical optimization through comparative evaluations and clinical outcome studies assessing diagnostic accuracy, consistency, efficiency, and sensitivity. Multi-center studies with reproducible methodologies across diverse practice settings will be essential for developing universally applicable recommendations and informing future teleradiology standards.

## Data Availability

No new data were generated or analyzed in this review article.

## Abbreviations

AAPM: American Association of Physicists in Medicine
ACR: American College of Radiology
AI: Artificial intelligence
CT: Computed tomography
DICOM: Digital Imaging and Communications in Medicine
GDPR: General data protection regulation
GPU: Graphics processing unit
GSDF: Grayscale standard display function
HIPAA: Health Insurance Portability and Accountability Act
IPsec: Internet protocol security
MFA: Multi-factor authentication
MRI: Magnetic resonance imaging
PACS: Picture Archiving and Communication Systems
QA: Quality assurance
RAM: Random access memory
UPS: Uninterruptible power supply
VPN: Virtual private network
